# Persistent Infection of Human Fetal Endothelial Cells with Rubella Virus

**DOI:** 10.1371/journal.pone.0073014

**Published:** 2013-08-05

**Authors:** Ludmila Perelygina, Qi Zheng, Maureen Metcalfe, Joseph Icenogle

**Affiliations:** Centers for Disease Control and Prevention, Atlanta, Georgia, United States of America; University of North Carolina School of Medicine, United States of America

## Abstract

Cardiovascular abnormalities are the leading cause of neonatal death among patients with congenital rubella syndrome (CRS). Although persistence of rubella virus (RV) in fetal endothelium has been repeatedly suggested as a possible cause of cardiovascular birth defects, evidence of the permissiveness of fetal endothelial cells to RV is lacking. In this study we evaluated the ability of RV to infect and persist in primary fetal endothelial cells derived from human umbilical vein (HUVEC). We found that wild type (wt) low passage clinical RV productively infected HUVEC cultures without producing cytopathology or ultrastructural changes. RV did not inhibit host cell protein synthesis, cell proliferation, or interfere with the cell cycle. Persistently infected cultures were easily established at low and high multiplicities of infection (MOI) with both laboratory and wt clinical RV strains. However, synchronous infections of entire HUVEC monolayers were only observed with clinical RV strains. The release of infectious virions into media remained at consistently high levels for several subcultures of infected HUVEC. The results indicate that macrovascular fetal endothelial cells are highly permissive to RV and allow slow persistent RV replication. The findings provide more evidence for the suggestion that vascular pathologies in CRS are triggered by persistent rubella virus infection of the endothelium.

## Introduction

Rubella virus (RV) is a single stranded RNA virus of positive polarity belonging to the genus *Rubivirus*, in the family *Togaviridae*. Postnatal rubella infection causes mild febrile illness accompanied by maculopapular rash and lymphadenopathy, while maternal infections during the first trimester of pregnancy often result in a combination of birth defects in newborns called congenital rubella syndrome (CRS) [1]. Although national immunization programs have led to the elimination or decline in incidence of postnatal rubella and CRS in many developed countries, approximately 100,000 CRS cases per year still occur worldwide [2].

RV can establish persistent infection in the developing fetus, where it continuously replicates and can induce multiple pathological changes [1,3,4]. However, overall organogenesis is not usually affected and CRS infants lack gross external and internal malformations. Even though RV can be isolated from multiple tissues and organs, only a limited number of cells show histological evidence of disease [5–7]. A prominent feature of fetal histopathology is noninflammatory damage to the endothelium of heart and blood vessels, which includes focal and obliterative lesions in large blood vessels and cellular damage in myocardium [6,8–11]. Vascular abnormalities can lead to a number of clinical manifestations with patent ductus arteriosus, pulmonary artery stenosis and septal and valve defects in heart being the most frequent [11,12]. Other clinical manifestations of congenital rubella, such as general growth retardation, deafness and neurodegenerative damage, may be due to vascular insufficiency leading to nutrient deprivation rather than a result of direct viral damage [4].

Most studies of fetal pathologies using human tissues were done in the 1960’s, and at that time the lack of molecular methods and reagents for RV detection precluded the precise localization of the sites of RV replication and persistence in congenitally infected fetuses. Nonetheless it was suggested that vascular pathologies in CRS cases were triggered by the persistent virus in endothelium [8,13,14]. One line of indirect evidence was an observed correlation between pathological changes and presence of RV antigen in placental blood vessels from CRS affected pregnancies [15]. Another line of indirect evidence was isolation of RV from diseased ductus arteriosus and umbilicus of CRS patients [9,16].

RV is a strictly human pathogen and animal models for CRS provide little useful information about the pathogenesis of congenital defects [4,17,18]. Several cell culture models using both continuous cell lines (e.g. Vero) and primary cells (e.g. fibroblast) persistently infected with RV have provided some information about molecular mechanisms of RV persistence and its effects on cell functions [19–22]. However, the infection of primary endothelial cell cultures by wtRV has not been reported to date.

To better understand the molecular mechanisms of vascular abnormalities in congenital rubella, we developed an endothelial cell culture model of rubella infection using primary cultures of fetal endothelial cells derived from human umbilical vein (HUVEC). We investigated the replicative characteristics of wtRV strains in HUVEC, the ability of clinical wtRV to establish persistent infection and the effects of RV infection on host protein synthesis and on proliferation of endothelial cells.

## Materials and Methods

### Cells and Viruses

HUVECs (3 different lots, each obtained from 20 pooled donors, Lonza, Allendale, NJ) were maintained in Endothelial Cell Growth Medium (Lonza, Allendale, NJ) and grown in flasks or plates coated with 0.1% gelatin (Sigma-Aldrich, St. Luis, MO) or fibronectin-coated chamber slides (BD Biosciences, Franklin Lakes, NJ). HUVECs were used between passages 3 and 5. A549 human lung carcinoma cells (ATCC #CCL-185) and Vero cells (ATCC #CCL-81) were maintained in Dulbecco's Modified Eagle Medium (high glucose) (Invitrogen, Carlsbad, CA) containing 5% FBS (Atlanta Biologicals, Lawrenceville, GA) supplemented with 50 µg/ml gentamicin (Invitrogen, Carlsbad, CA). Uninfected cells were cultivated in a humidified atmosphere with 5% CO_2_ at 37° C, whereas rubella infected cells were maintained at 35° C. The laboratory strain F-Therien was originally obtained from Dr. Frey’s Laboratory. F-Therein is a derivative of wt clinical strain Therein, which was three times plaque purified on Vero cells and selected for large plaques and high virus yield in Vero cells [23]. Clinical isolates RVi Dezhou.CHN 02 (RV-Dz, genotype 1E) and RVi Seattle.USA 16.00 (RV-WA, genotype 2B) were isolated at the CDC Rubella Laboratory. All RV strains were propagated in Vero cells and titered on Vero cells by immunocolorimetric plaque assay [24]. The titer was expressed as plaque forming units (pfu) per ml.

### Preparation of High Titer Virus Stocks

Vero cells were grown to high density on FibraCel discs in single-use 500 ml bottles using a FibraStage system (New Brunswick Scientific, Edison, NJ) and infected with RV at MOI=0.01 pfu/cell. Culture media were collected daily from 3 dpi to 9 dpi, centrifuged at 1500 x g for 20 minutes to remove cell debris and then concentrated by tangential flow filtration using a Biomax-300 cassette filter (Millipore, Billerica, MA). To partially purify virus, the concentrated supernatants were diluted 10-fold with Minimum Essential Medium (Invitrogen, Carlsbad, CA) and re-concentrated. The procedure was repeated twice to achieve ~100-fold purification. The titers of concentrated virus stocks were 2-3x10^8^ pfu/ml. To be used for a mock control, the spent medium from uninfected Vero cells were concentrated and purified using the same procedure. The viral stocks (passage 7) were stored at -80° C in aliquots.

### Growth curve analysis

Cells were seeded into 48-well cell culture plates at 1x10^5^ cells/well and infected with RV at MOI of 5 (single-step growth curve) or 0.05 (multistep growth curve) the following day. After 1-hour adsorption at 35° C, the monolayers were washed 3 times with Hank’s Balanced Salt Solution (HBSS) and overlaid with 0.5 ml of fresh media. Supernatants and cells were collected after 10 minutes incubation (0 hpi) and subsequent samples were collected at later times. Cell lysates were prepared by adding 0.5 ml media to monolayers followed by 3 cycles of freeze-thaw. Virus was titered on Vero cells in duplicate.

### Cell Cycle Analysis by Flow Cytometry

HUVECs were serum-starved overnight in 0.2% FBS and then mock-infected or infected with RV-Dz at MOI=10. The cells were collected at 1 dpi by trypsinization, washed with ice-cold PBS, resuspended in 200 μL of PBS and added dropwise into 4 ml of ice-cold 70% ethanol. After overnight fixation (-20° C) the cells were centrifuged at 800 x *g* for 10 minutes, resuspended in 0.5 ml PBS containing 40 µg/ml propidium iodide (Sigma-Aldrich) and 100 µg/ml RNase (Invitrogen) and incubated at 37° C for 30 minutes. Total DNA content was analyzed using a LSRII flow cytometer and FACSDiva 5.01 software (BD Biosciences, Franklin Lakes, NJ).

### RNA Extraction and Quantitation

Cells were seeded into 6-well cell culture plates at 4x10^5^ cells/well and mock-infected or infected with RV-Dz at MOI of 5. RNA was isolated using RNAeasy Mini kit (Qiagen) according to the manufacturer’s instructions. RNA concentration was measured with NanoDrop spectrophotometer (Thermo Scientific, Rockford, IL). RT-qPCR was performed on a 7500 real-time PCR system (Applied Biosystems, Foster, CA) using Quantifast Multiplex RT-PCR kit (Qiagen). RNA (100 ng) was amplified using the following primers and probes: for genomic rubella RNA, RV195F and RV323R primers and RVP3 probe [25], for the glyceraldehyde-3-phosphate dehydrogenase (GAPDH) gene, GAPDH-F (5’-GAAGGTGAAGGTCGGAGTC-3’) and GAPDH-R (5’-GAAGATGGTGATGGGATTTC-3’) primers and GAPDH-P (Cy5-CAAGCTTCCCGTTCTCAGCC-BHQ2) probe. Since the difference of the slopes of the calibration curves for RV and GAPDH RNA were less than 0.1, the data were analyzed with the comparative threshold cycle (ΔΔ C_T_) method. Data are presented as a fold change of genomic RNA amount normalized to GAPDH and relative to the genomic RNA amount at 4 hpi, when the lowest amount of viral RNA was detected.

### Whole Genome Sequencing

A detailed strategy for a whole genome sequencing of rubella virus has been described [26].

### Western Blot Analysis

Cell monolayers were washed 3 times with ice-cold PBS and then proteins were extracted with RIPA buffer (Thermo Scientific, Rockford, IL) supplemented with Halt protease cocktail (Thermo Scientific, Rockford, IL). Protein concentration in the extracts was determined by BCA assay (Thermo Scientific, Rockford, IL). Equal amounts of total protein (15 μg/well) for each sample were separated by 4-12% nonreducing NuPage (Invitrogen, Carlsbad, CA) using MOPS running buffer, blotted onto nitrocellulose membrane and processed using SNAP i.d. ® Protein Detection System (Millipore, Billerica, MA) according to the instrument manual. Briefly, membranes were blocked in 0.5% milk-PBST (PBS-0.1% Tween-20) for 1 minute and then incubated with primary antibodies (1:1000 dilution in 0.5% milk-PBST) for 15 minutes. The following rubella-specific monoclonal antibodies were used: CDC anti- E1 (produced by the CDC core facility), anti-E2 (Meridian Life Science, Memphis, TN) and anti-capsid (Abcam, Cambridge, MA). After washing 3 times with PBST the blots were incubated with horseradish peroxidase-conjugated anti-mouse IgG (1:1000 dilution in 0.5% milk-PBST) (Thermo Scientific, Rockford, IL) for 15 minutes. The signal was developed using ECL-plus detection reagents (GE Healthcare, Piscataway, NJ). After removing bound antibodies with Restore Western Stripping Buffer (Thermo Scientific, Rockford, IL) the blots were re-probed with mouse HRP-conjugated β-actin MAb (Sigma-Aldrich, St. Louis, MO) to verify equal protein loading.

### Immunofluorescence analysis (IFA)

HUVEC cultured on fibronectin-coated chamber slides (BD Biosciences, Franklin Lakes, NJ) were infected with RV at MOI=5 or RV-infected HUVECs were plated onto chamber slides when the infected cultures were passaged. At different times postinfection the monolayers were rinsed with PBS, fixed with -20° C methanol for 10 minutes and blocked for 1 hour at room temperature in 10% normal goat serum-PBST. The cells were stained with rubella specific MAb or rabbit polyclonal antibody against von Willebrand factor (vWF) (Sigma-Aldrich, St. Louis, MO) for 1 hour at room temperature, followed by incubation with anti-mouse IgG-Alexa488 or anti-rabbit IgG-Alexa546 antibodies (Molecular probes, Invitrogen) and counterstaining with DAPI (Molecular probes, Invitrogen). Cells were mounted with fluorescence mounting medium (DakoCytomation, Carpinteria, CA). Images were acquired using a Zeiss fluorescent microscope AxioImager.A1 equipped with AxioCamMRc5 digital camera. To estimate the percentage of infected cells, positively stained cells and nuclei were counted in at least four microscopic fields (~100 cells/field).

### Cell proliferation assay

HUVEC were mock-infected or infected with RV-Dz at MOI=10 in a T25 culture flask. The following day the infected or mock-infected cells were simultaneously plated into multiple wells of 6-well plates with a low seeding density of 5x10^4^ cells/well and cultured for 2-5 days without passaging. Daily, the mock-infected and infected cells were collected by trypsinization from duplicate wells and counted using a Scepter cell counter (Millipore, Billerica, MA). Growth curves were generated by plotting the number of cells in a well against time in a culture. After counting, the mock-infected and RV- infected cells were plated onto chamber slides, and the next day were immunostained by IFA with capsid MAb (Abcam, Cambridge, MA) and DAPI (Molecular Probes, Invitrogen) to quantitate infected cells and mitotic figures. The mitotic indexes were calculated as % of cells with mitotic figures.

### Non-radioactive metabolic labeling of total cellular proteins

Proteins were labeled using Click-iT L-azidohomoalanine (AHA) metabolic protein-labeling reagent (Invitrogen, Carlsbad, CA). At various times postinfection the cell monolayers were washed with methionine-free RPMI 1640/2% FBS (Invitrogen, Carlsbad, CA) for 30 minutes and then incubated with methionine-free RPMI 1640/2% FBS supplemented with 25 µM AHA for 1.5 hours. The monolayers were washed thrice with PBS and then proteins were extracted with RIPA buffer and quantified by BCA assay (Thermo Scientific, Rockford, IL). Newly synthesized proteins were detected with biotin-alkyne detection reagent and Click-It protein reaction buffers (Invitrogen, Carlsbad, CA) according to manufacturer’s protocol. Briefly, each sample was mixed with biotin-alkyne and incubated at room temperature for 20 minutes. After extraction of residual reaction components with chloroform, proteins were precipitated with methanol, and then equal amounts of proteins (5 μg/lane for detection of AHA-labeled proteins and β-actin and 20 μg/lane for capsid detection) were separated by 4-12% reducing NuPage (Invitrogen, Carlsbad, CA) using MOPS running buffer and transferred onto a nitrocellulose membrane and processed manually. The membrane was blocked with 5% milk-PBST for 1 hour, incubated with streptavidin-poly-HRP (Vector Labs, Southfield, MI) and then the signal was detected using ECL-plus detection reagents (GE Healthcare, Piscataway, NJ).

### Transmission electron microscopy (TEM)

Cells were infected with RV at an MOI of 50. Cell culture pellets were collected at 24 hpi or 17 dpi and fixed with 2.5% glutaraldehyde. Pellets were postfixed in 1% osmium tetroxide followed by deionized water rinses and *en bloc* staining with 4% uranyl acetate. After rinsing the specimen with deionized water, the pellets were dehydrated in an alcohol series and infiltrated with acetone. Three ratios of acetone to resin (2:1, 1:1 and 1:2) were used prior to four exchanges of 100% resin (Epon substitute and Araldite). Polymerization was completed overnight at 60° C. Thin sections were cut and stained with uranyl acetate and lead citrate before viewing sections with the electron microscope (Tecnai Spirit, FEI).

### Statistical analyses

The two-way analysis of variance (ANOVA) test with the Bonferroni posttests was used to compare differences between virus titers produced by three cell lines at different times postinfection. A *P* value of <0.05 was considered significant. Statistical analyses were performed using the GraphPad Prizm 5 software (GraphPad Software, San Diego, CA).

## Results

### RV Replication in Endothelial Cells

Since pathologic lesions are often observed in large elastic blood vessels of CRS patients including umbilical vein [14], we used primary cultures of endothelial cells derived from umbilical vein to examine the susceptibility of fetal endothelial cells to RV. To ensure that HUVECs retain their specific properties, cells were always used for experiments before they reached passage 6 [27]. To evaluate the ability of fetal endothelial cells to support RV replication, we performed single-step and multistep growth curve analysis by infecting HUVECs with RV-Dz at an MOI of 5 and 0.05, respectively, and measuring accumulation of infectious rubella virions in the culture media. This isolate was selected based on its genotype (1E), which is one the most frequently reported globally [28]. For comparison, we carried out growth assays in Vero cells because RV replication in this cell line has been investigated in detail [29,30]. A second comparison cell line A549 was chosen because of its human origin and its intact IFN system.

**Figure 1:**
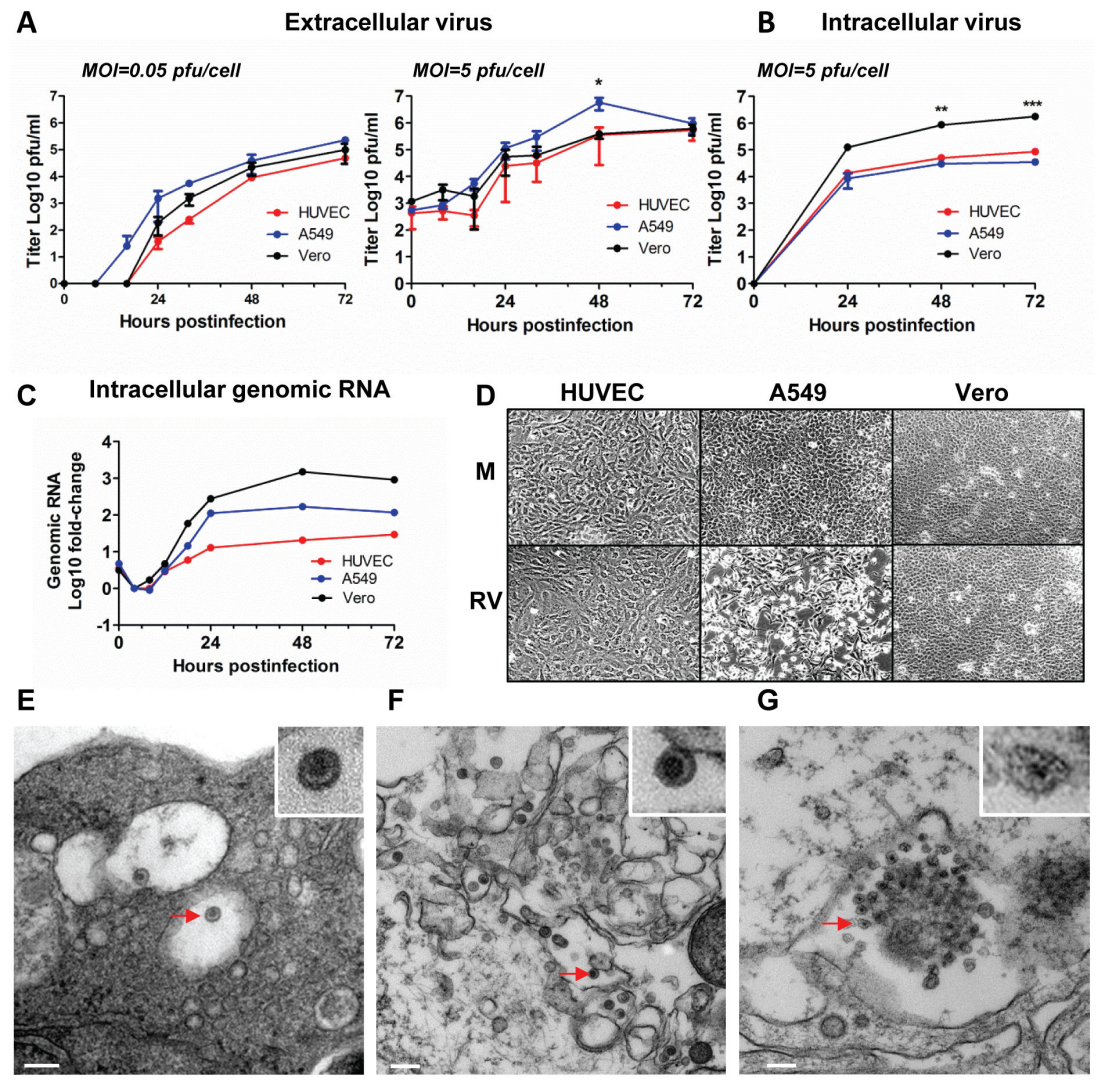
Productive infection of HUVEC with low passage wtRV. (**A**–**B**) Kinetics of RV replication in HUVEC, Vero and A549 cells. Cells were infected with RV-Dz at an MOI of 0.05 or 5. Cell culture supernatants (A) or cell lysates (B) were titered in duplicate on Vero cells. Data are presented as a mean value +/- standard deviation of two independent experiments each performed in duplicate. The data were analyzed by two-way ANOVA with the Bonferroni posttests for correcting for multiple comparisons (*, P<0.05; **, P<0.01; ***, P<0.001). (**C**) Quantitation of intracellular rubella genomic RNA. HUVEC, Vero and A549 cultures were infected with RV-Dz at an MOI of 5. Genomic RNA was quantitated by RT-qPCR. GAPDH mRNA was used for normalization in the comparative threshold cycle method. Data are presented relative to the genomic RNA amount at 4 hpi. The results represent the mean of at least two independent experiments each done in duplicate. (**D**) Phase contrast pictures of cells at 5 dpi either mock infected or RV-Dz infected at MOI=5. Note cytopathic effect of wtRV in A549. (**E**) Representative images of rubella virions observed by TEM in HUVEC infected with RV-Dz at MOI=50 at 24 hpi. (**F**) Representative images of rubella virions and (**G**) replicative complexes observed by TEMin Vero cells infected with RV-Dz at MOI=50 at 24 hpi. Inserts represent enlarged images from the replicative complex and virions that are marked with the red arrows. Bars, 100 nm.

RV growth kinetics in HUVECs and Vero cells were comparable (Figure 1A). The release of newly synthesized virions was first detected at 24 hpi at both MOIs. Results of multistep growth analysis (MOI=0.05) showed that RV can spread effectively in HUVEC monolayer. Results of single step growth analysis (MOI=5) showed that virus production reached the maximum value of approximately 5x10^5^pfu/ml by 48 hpi in both cell types. Given that there were 10^5^ cells/well plated, the production of extracellular virus in HUVEC and Vero cells was estimated to be ~5 pfu/cell daily. Initially, RV replication in A549 cells was more efficient than in HUVECs and Vero but decreased after peaking at 48 hpi at high MOI (Figure 1A). CPE in a form of cell rounding and detachment from the monolayer was evident in A549 cells at 72 hpi followed by massive cell death after 5 dpi, whereas no evident CPE was observed in HUVEC and Vero (Figure 1D). We were unable to subculture the infected A549 cells.

RV has been shown to egress with different efficiencies from different cell lines [31]. To assess RV export from infected HUVECs, we compared the infectious virus titers in supernatants and cell lysates. In infected Vero cells, the titer of extracellular virus was approximately 10% of the intracellular titer, whereas in both human cell cultures the reverse situation was observed (Figure 1A-B). These data suggest that egress of wtRV is more efficient from HUVEC and A549, than from Vero cells.

The kinetics of genomic RNA replication was studied by relative quantitation of RV genomic RNA in infected cells by real-time RT-qPCR (Figure 1C). After the 12-hour eclipse period, only a 20- to 30-fold increase over the amount of viral RNA at the eclipse phase was observed at 24 hpi and thereafter in infected HUVEC. These results suggest that slow replication kinetics of rubella genomic RNA was mainly responsible for the low level of virus production in endothelial cells as it has been shown for other cell types [30,32]. Approximately 10 times more RNA was produced in A549, which correlated well with higher virus yield relative to HUVEC (Figure 1A). In HUVEC, RV RNA does not accumulate in the cytoplasm after synthesis; presumably it is packaged and continuously released into media, where infectious particles accumulate. Consequently, infectious titers in the media from 24 to 72 hours post infection were increased more efficiently than quantities of genomic RNA in cytoplasm at the same time. Vero cells accumulated 2 logs more intracellular genomic RNA but only 1 log more infectious particles by 48 hpi compared to HUVEC (Figure 1B). Intracellular accumulation of large quantities of genomic RNA has not been observed in Vero cells infected with laboratory strains adapted to Vero culture [30,32]. These data suggest that Vero cells are less efficient in both assembly and release of wild type infectious virus than HUVEC.

Additional evidence of low level of intracellular RV and efficient export in HUVEC was obtained by transmission electron microscopy. Very few intracellular and extracellular viral particles were observed among the infected HUVEC cultures after 24 hpi and replication complexes were not readily observable (Figure 1E). By contrast, replication complexes and larger numbers of intracellular particles were found in Vero cells (Figure 1F-G), which is in agreement with the results of our growth experiments and published reports by others [33]. After infection with low passage clinical strains HUVEC did not exhibit changes in morphology of mitochondria and rough endoplasmic reticulum or re-distribution of mitochondria, as observed by others in Vero cells infected with RV lab strains [34].

**Figure 2:**
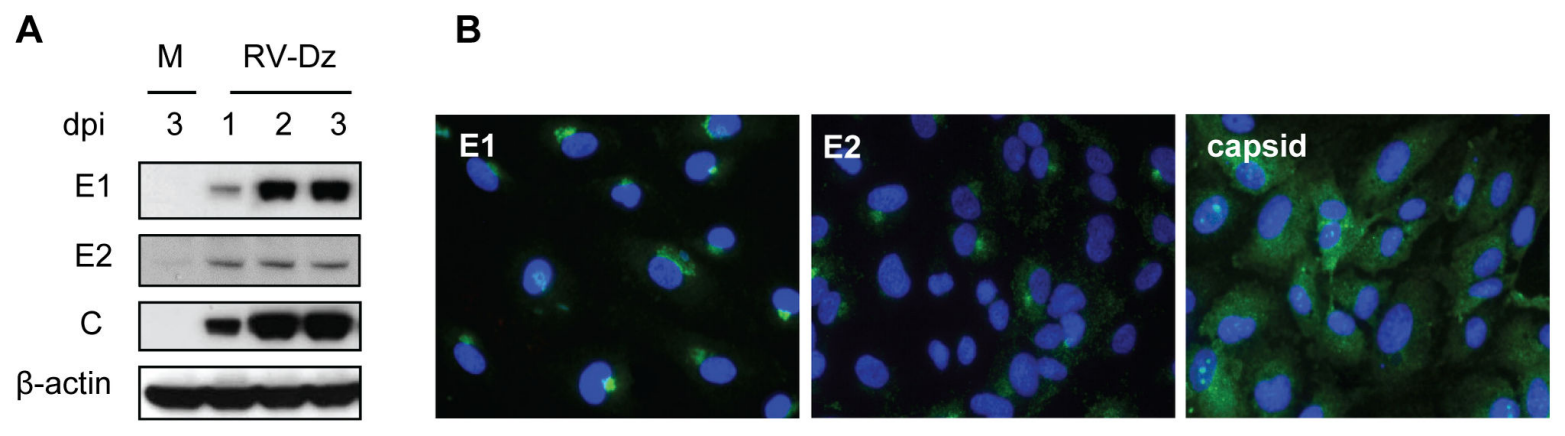
Expression of viral structural proteins in infected HUVEC. (**A**) Kinetics of viral protein synthesis. HUVEC were mock infected (M) or infected with RV-Dz at an MOI=5. Proteins were separated by 4-12% NuPage gel, either nonreducing (E1, C, β-actin) or reducing (E2), and then the blots were probed with rubella E1, E2 and C-specific MAb and β-actin MAb. (**B**) Spatial distribution of E1, E2 and C proteins in infected cells. HUVECs were infected with RV-Dz at an MOI=5 on chamber slides and processed for indirect immunofluorescence at 2 dpi using E1, E2 and capsid-specific MAb. Nuclei were counterstained with DAPI.

We also examined expression of RV structural proteins in infected HUVEC cultures. E1, E2 and capsid were detectable by Western blot and IFA as early as at 1 dpi (Figure 2). The intracellular distribution of the proteins was similar to that seen in other cell types: E1 and E2 were apparently localized in trans-Golgi (a tight halo around nuclei), while capsid was diffusely distributed in cytoplasm (Figure 2B). These data also indicate that infection of HUVECs with MOI=5 results in practically synchronous infection: 82%, 91% and 95% of cells were antigen positive at 1, 2, and 3 dpi, respectively. The synchronous infection of HUVEC monolayers was also observed after infection with RV-WA clinical isolate at MOI=5 (data not shown).

Collectively, the data in figures 1 and 2 demonstrate that RV can productively infect and spread in human fetal endothelial cells without producing significant cytopathology.

### Effects of RV Replication on Global Protein Synthesis

**Figure 3:**
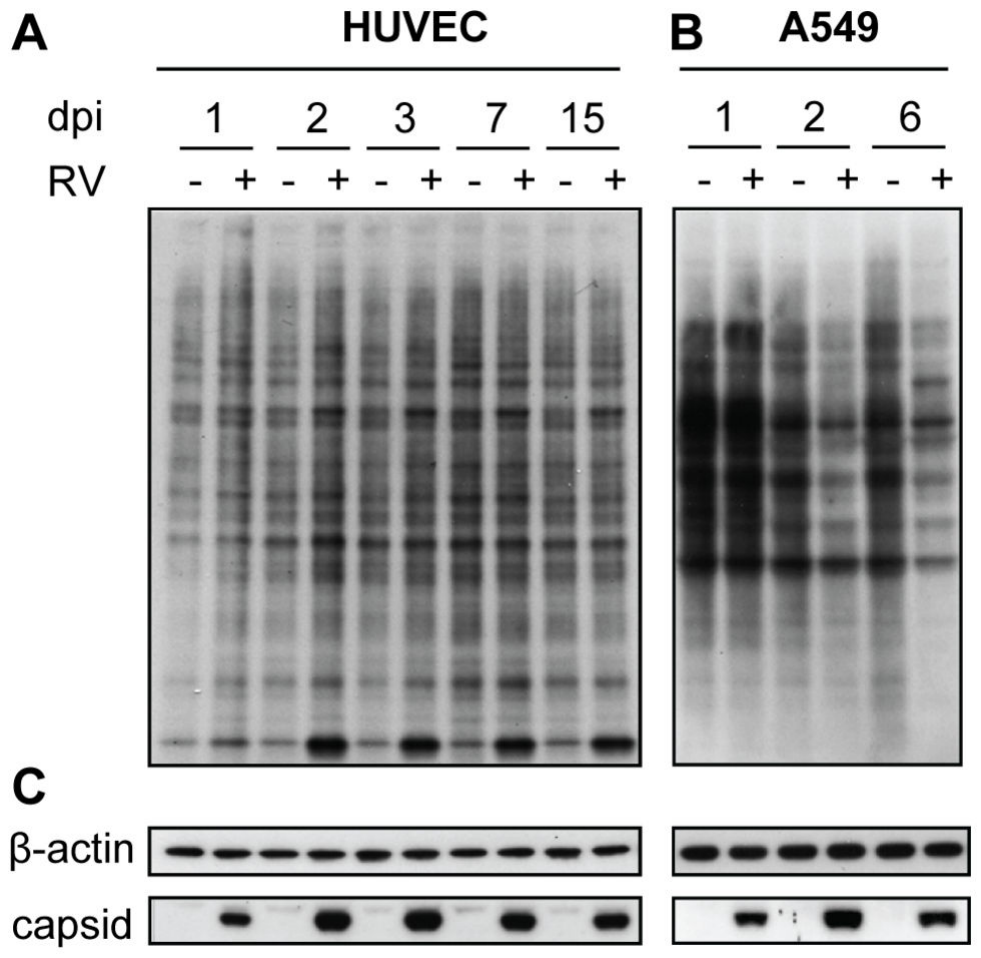
Lack of global cellular protein synthesis shut-off in infected HUVEC. HUVEC and A549 cells were mock infected or infected with RV-Dz at MOI=5. The cultures were metabolically pulse-labeled with non-radioactive protein-labeling reagent AHA at the indicated times postinfection. Equal amounts (5 μg/lane for detection of AHA-labeled proteins and β-actin and 20 μg/lane for capsid) of each sample were separated by 4-12% NuPage and blotted onto nitrocellulose membrane. (**A**–**B**) Blots were probed as described in Material and Methods to reveal newly synthesized proteins. (**C**) The blots were also probed with β-actin MAb to demonstrate equal protein loading and with capsid MAb to confirm RV infection. Representative results of two independent experiments are shown.

Depending on the cell type, RV has been shown to induce different degrees of cellular protein synthesis shutoff [35]. To assess total protein synthesis in HUVEC during RV infection, metabolic pulse-labeling experiments followed by Western blot analysis were carried out with the HUVEC cultures infected with RV-Dz at an MOI of 5. No inhibition of total cellular protein synthesis was observed in the infected endothelial cultures for up to 15 dpi (Figure 3A). Since the protein synthesis shutoff has only been demonstrated in non-human cells (Vero, BHK, and RK13) [35], it was unclear whether the inability of RV to interfere with protein synthesis in HUVEC is specific to HUVEC or is characteristic of human cells. To differentiate between these possibilities we performed metabolic pulse-labeling experiments with A549 infected with RV-Dz at an MOI of 5. The global protein synthesis was unchanged at 1 dpi, but clearly reduced by 2 dpi and later times in the infected A549 cells (Figure 3B). This reduction was not likely due to cell death because we and others did not observed any CPE or cell death in A549 infected monolayers at 2 dpi as assessed by IFA staining for activated caspase-3 (data not shown and [36]). These data demonstrate that the ability of RV to interfere with host protein synthesis is not characteristic of all human cell types. The data also confirm that the recently developed method of non-radioactive metabolic labeling of newly synthesized proteins [37] is sensitive enough to detect changes in protein synthesis in virally infected cells.

### Effects of RV Infection on HUVEC Proliferation

**Figure 4:**
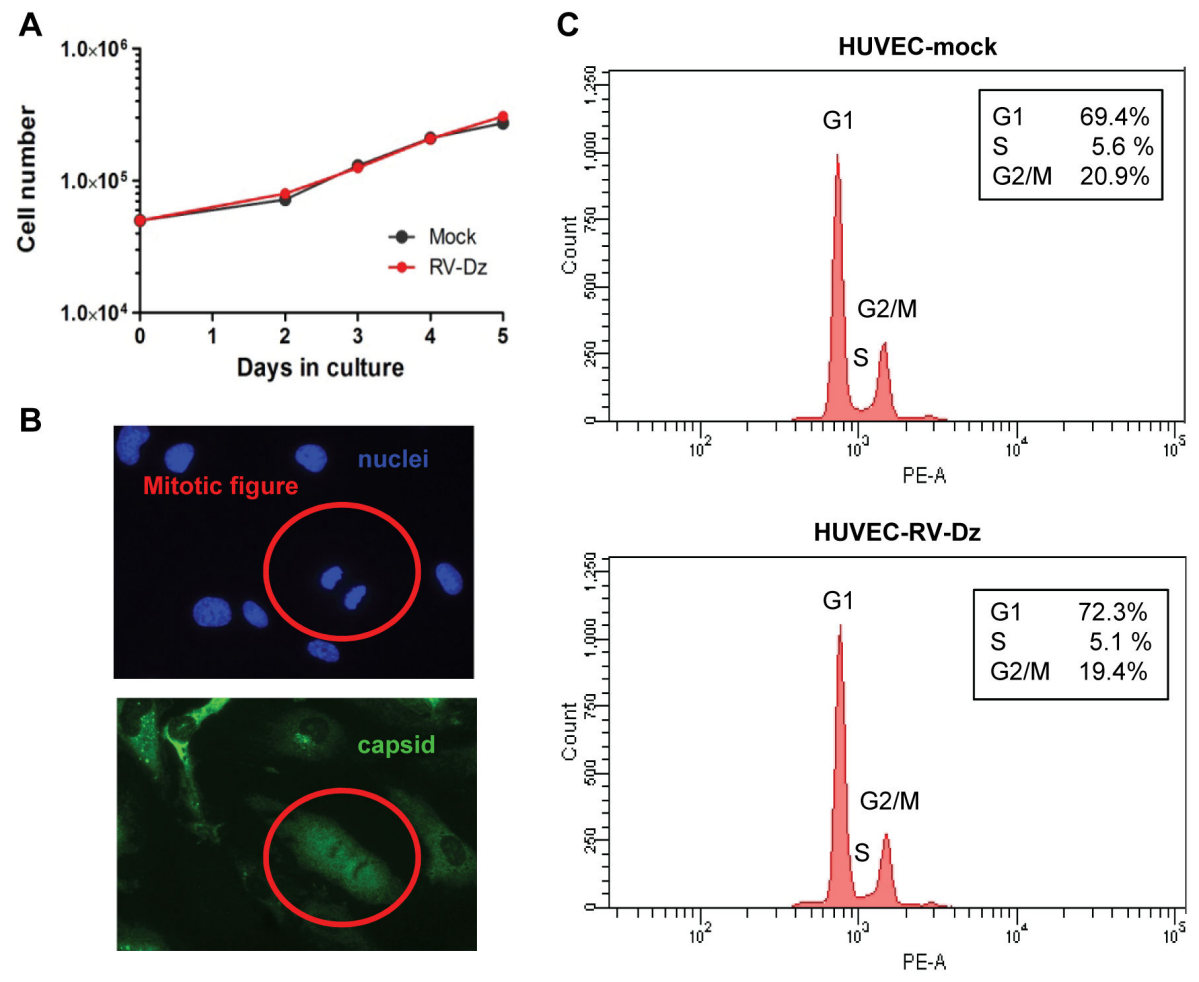
Effects of RV infection on cell proliferation and mitosis. (**A**) Growth curves of mock-infected and RV-infected HUVECs. HUVEC were mock infected or infected with RV-Dz at MOI=10 and then counted daily. The data are results of 2 independent experiments each performed in duplicate. (**B**) Mitosis in infected HUVEC. At 2 dpi the mock infected and infected HUVEC (RV-Dz, MOI=10) were immunostained by IFA with capsid MAb and DAPI to quantitate infected cells and mitotic figures. Mitotic indexes (MI) were calculated as % cells with mitotic figures in two duplicate wells. Note RV-antigen positive mitotic cell in red circle. (**C**) Cell cycle analysis of infected HUVEC. Serum-starved HUVECs were mock-infected or infected with RV-Dz at MOI=10. Histograms of cell cycle analysis at 1 dpi show DNA content of propidium iodide-stained cells by flow cytometry and % of cells in each phase of the cell cycle. The representative results of two independent experiments are shown.

To determine the effect of rubella infection on growth of endothelial cells, we used the cell proliferation assay (see Material and Methods) to compare growth curves and mitotic indexes of the mock-infected cultures and cultures infected with RV-Dz at MOI=10; virtually all cells were positive for RV antigen at 1 dpi (data not shown). The growth rates of both cultures were almost identical (Figure 4A). The mitotic indexes of mock-infected and infected cells were comparable (0.18% and 0.16%, respectively). Most cells in the infected cultures remained rubella antigen positive for the duration of the experiment (5 days) and the proportion of infected cells did not change indicating RV infected cells proliferate. Moreover, cells with mitotic figures stained positive for RV capsid (Figure 4B) demonstrating that infected cells were undergoing mitosis. These data indicate that RV replication does not interfere with mitosis and does not inhibit cell proliferation.

We also quantified cells in each phase of the cell cycle in RV-infected and mock-infected cultures by flow cytometry after staining cellular DNA with propidium iodide. Both cultures displayed comparable distributions of cells in each phase (Figure 4C) supporting the hypothesis that RV does not affect cell cycle progression in HUVEC.

### RV Persistence in Endothelial Cells

**Figure 5:**
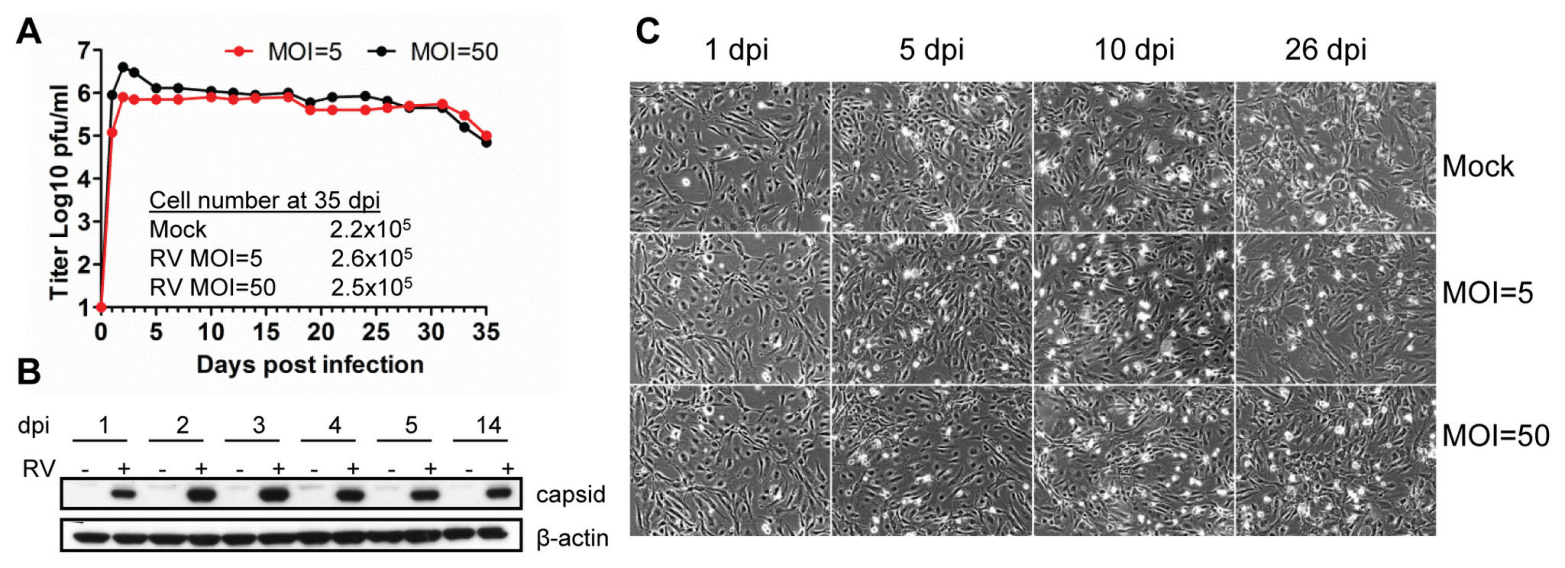
RV persistence in HUVEC. (**A**) Growth curves of RV-Dz at different MOI. Media were collected every 2-3 days and the extracellular viruses were titered on Vero cells in duplicate. The adherent cells on the flasks at 35 dpi were collected by trypsinization into 4 ml of media and counted (cells/ml) using a Scepter cell counter. The representative results of two independent experiments are shown. (**B**) Western blot analysis of RV capsid protein expression in persistently infected HUVEC. The blot was re-probed with β-actin MAb to confirm equal protein loading. (**C**) Phase contrast images of cells at different times postinfection showing the lack of CPE in HUVEC persistently infected with RV-Dz strain.

To evaluate the ability of RV to persist in endothelial cells, we monitored virus production in RV-Dz infected HUVEC cultures, which were maintained without splitting until senescence (35 dpi). In addition to MOI of 5, HUVECs were also infected with RV-Dz at MOI of 50 to ensure synchronous infection of all cells in the monolayer and to match the TEM experiments. The virus production curves were similar for MOIs of 5 and 50. Virus production reached a maximum at 2 dpi and remained at approximately the same level thereafter for both MOIs (Figure 5A). Capsid protein expression also reached a maximum at 2 dpi and remained at approximately the same level up to 14 dpi (Figure 5B). Despite the virus replication, no differences in cell morphology were observed in the infected monolayers relative to mock-infected cells at any time during the experiment (Figure 5C). The numbers of cells in the mock-infected and infected cultures were comparable at 35 dpi (Figure 5A) and virtually all cells were E1-antigen positive as determined by IFA (data not shown). These data further confirmed the lack of inhibition of HUVEC cell growth by RV infection. Additionally, no differences in ultrastructure between RV-Dz- and mock-infected HUVEC cultures were seen by TEM at 17 dpi (data not shown). The whole genome sequences of the RV-Dz stock and the viruses isolated from the persistent cultures at 32 dpi were identical (GenBank acc. # KF201674).

Infection of HUVECs with a different clinical isolate, RV-WA, at MOI=5 also resulted in persistent culture, in which virus production was at the same level as in the RV-Dz cultures for the duration of the experiment and all cells were stained RV-antigen positive at14dpi (data not shown). Moreover, persistent cultures were obtained after infection of HUVEC with two additional passage 2 clinical isolates (genotypes 2B and 1G) at MOI=0.05. IFA staining for E1 confirmed that all cells in the persistent cultures were infected (data not shown). Thus, basic characteristics of persistence with clinical isolates in HUVEC were shown with a total of four clinical isolates. Experiments with two different lots of HUVEC produced similar results (data not shown). Collectively, the data indicate that fetal endothelial cells can sustain persistent infection with most wtRV regardless of MOI.

**Figure 6:**
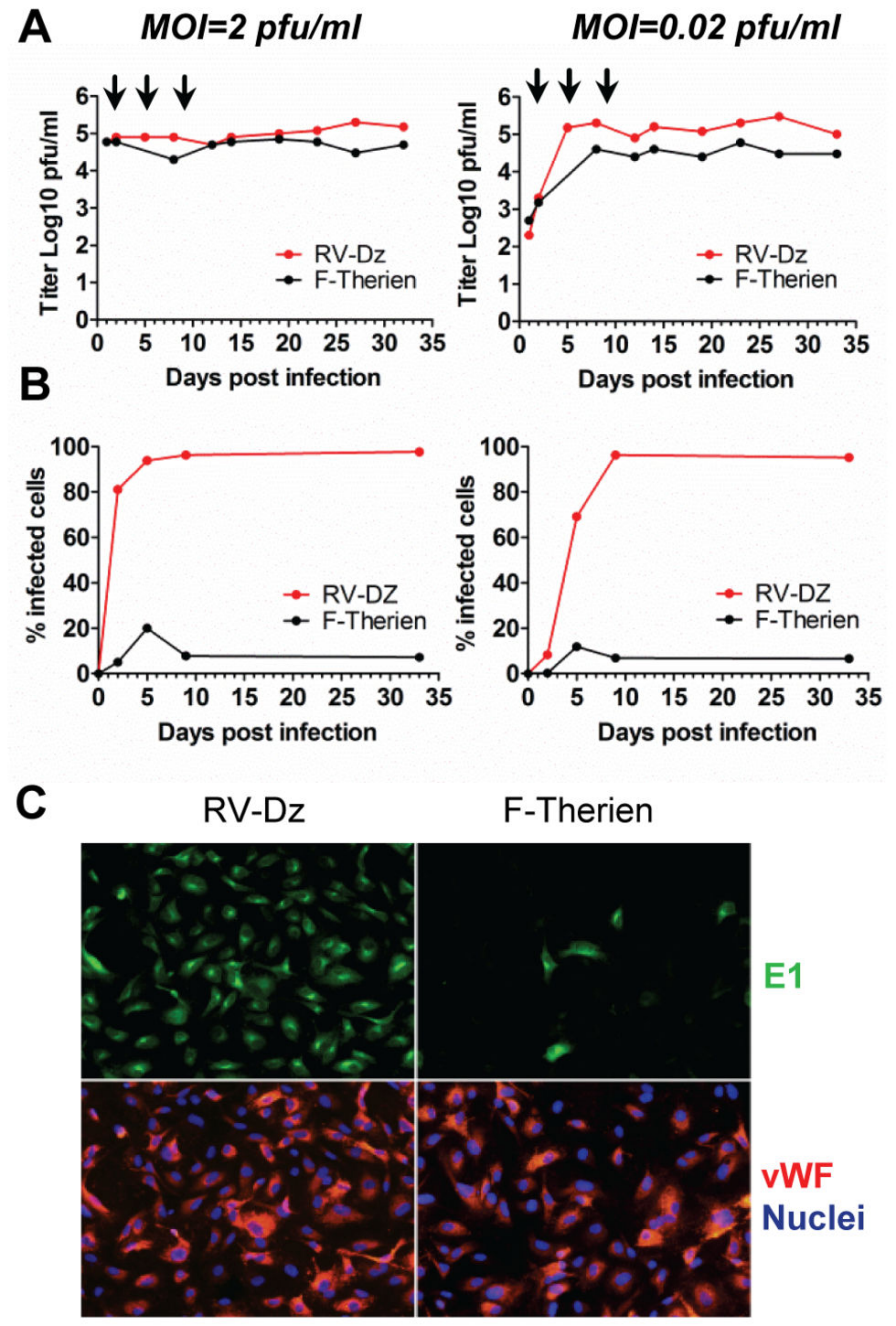
RV persistence after sequential passages of infected HUVEC. Cells infected at low and high MOI were passaged 1:4 at 3, 5 and 9 dpi (indicated by the arrows). (**A**) Media were changed every 2-3 days and titer of extracellular virus was determined by titration on Vero cells. The data are representative results of 2 independent experiments. (**B**) After each passage, the number of the infected cells was determined by counting E1-positive cells after IFA staining of infected cells for E1, endothelial cell marker (vWF) and counterstaining for nuclei (DAPI). (**C**) The representative results of IFA staining of the infected cells immediately after the third passage.

The ability of RV to persist in endothelial cells during sequential passages of infected cultures was compared with that of plaque purified F-Therien, one of the best-characterized laboratory strains, because the ability of F-Therien to persist in some cell cultures has been previously demonstrated [21,22]. HUVECs were able to sustain persistent infection with RV-Dz and F-Therien following three passages when infected at either high or low MOIs (Figure 6A). Most cells remained positive for endothelial cell marker vWF (Figure 6C). Unlike clinical isolates, which infected all cells in a monolayer, F-Therien infected a small fraction of cells at both MOIs (Figure 6B-C). Infections with larger virus doses (MOI of 20 and 200) resulted only in minor increases of a number of F-Therien infected cells (data not shown). Possibly, plaque purification and the long-term passage of F-Therein in Vero cells (epithelial origin) has resulted in changes in endothelial cell tropism as has been reported for cytomegalovirus (CMV) after *in vitro* propagation of clinical strains in fibroblasts [38].

Despite the fact that only about 5% of cells in HUVEC persistent cultures were infected with F-Therein, the yield of virus was comparable to RV-Dz infected cultures (Figure 6). This indicates that F-Therein was able to replicate in HUVEC with higher yields/cell than that of wt virus, RV-Dz (Figure 6A-B). Originally, F-Therein strain was plaque-purified and selected for high replication rates; it produces at least 1 log more infectious virions in Vero cells than any other rubella strain studied ( [23]; our unpublished data). Highly efficient replication of F-Therein was linked to the specific mutations in the F-Therein genome not found in wt strains and to synthesis of elevated levels of non-structural replicase proteins [39]. Thus, although F-Therein was able to establish persistent infection in HUVECs, the characteristics of viral persistence for F-Therein are different from that of low passage clinical RV strains making this laboratory strain unsuitable for studying wt rubella persistence in endothelial cells. 

## Discussion

In this study we investigated RV persistence in primary fetal endothelial cells since this cell type is involved in pathogenesis in congenitally infected fetuses and abnormal endothelium is found in CRS cases. We found that this system was significantly different from other systems (e.g. laboratory RV strains in Vero cells) and may be a good *in vitro* model for investigation of molecular mechanisms of RV persistence.

Growth curve experiments combined with detection of rubella structural proteins and viral particles unequivocally showed that low passage clinical RV can productively infect and efficiently spread in primary human endothelial cells without producing cytopathology. To our knowledge this is the first report demonstrating endothelial cell tropism of RV. Although other investigators reported the ability of RV to infect an endothelial cell line ECV304 [40], this cell line has been proven to be a cross-contamination with a human bladder cancer cell line T24/83, which is not of endothelial origin and thus not suited to study endothelial cell biology [41].

Some characteristics of RV replication in HUVEC including kinetics of genome replication, structural proteins synthesis and production of infectious particles were comparable to those observed by others in commonly used cell lines [30,42]. Additionally, as in other primary cell cultures [19,43,44], RV infection in endothelial cells had no discernible cytopathic effect. However, at least four characteristics of RV infection in endothelial cells differ from those reported for non-endothelial cell types.

1. RV infections at MOI≥10 produced synchronously infected HUVEC cultures in which virtually all cells were RV-antigen positive at 1 dpi. This is in contrast to other cell types (e.g. Vero, BHK-21 and human fibroblasts), in which only a fraction of cells in a culture (10-50%) were infected initially following virus adsorption (asynchrony), even at high MOIs [30,35,45,46]. A dependence of rubella replication on a cellular component present in fluctuating quantities during the cell cycle was suggested as a possible explanation for asynchronous RV infection [30,35]. Asynchrony of RV infection could result from an inefficient entry of extracellular virions due to either low receptor density in the cell types previously tested or poor RV-receptor interactions in cells of non-human origin. In any case, such models of asynchrony do not seem necessary for wtRV infection of HUVEC since efficient virus penetration and synchronous infection was observed.
2. Infectious RV virions were effectively released from HUVEC. About one log more infectious RV-Dz was recovered from extracellular medium compared to intracellular virus (Figure 1B). In contrast, the titers of cell-associated wtRV were equal or greater than the viral titers in the culture media in non-human cell types commonly used to study wtRV replication: BHK-21, RK-13 [47] and Vero cells (Figure 1 B). Although efficient RV release from Vero cells has been reported [31], it is true only for Vero cell-adapted RV strains, not clinical isolates (our unpublished results). Virus egress was equally efficient from A549 (Figure 1B), another human cell culture tested here. Moreover, attempts to identify intracellular virus in tissues of CRS fetuses were mostly unsuccessful indicating efficient RV secretion from cells *in vivo* [48,49]. Taken together these observations suggest that the wtRV egress mechanism is fine-tuned to human cells or possibly to particular human cell types.
3. Infected HUVEC have been observed to secrete infectious viral particles at 2-3 logs higher titers compared to other primary cell cultures [20,43,44,50,51]. The difference in the proportion of infected cells in HUVEC (>95%) and these other primary cell cultures (ranging from 5 to 70% [43,44,46,51]) is not sufficient to explain the dramatic differences in the titers of secreted RV. One explanation is that some cellular factors essential for RV replication could be present in abundance in HUVEC but in limited quantities in other cell types. More efficient secretion of viral particles from HUVEC might be involved since endothelium is a very active secretory organ [52].
4. RV interference with growth properties of HUVEC was negligible and neither global protein synthesis nor cell proliferation nor cell cycle was affected in infected HUVEC. This is in contrast to the numerous studies documenting RV inhibitory effects on cell growth and proliferation of continuous cell lines and primary fibroblast cultures derived from fetal organs [19,20,53–56]. It appears that RV might exert different effects on cellular growth and metabolism depending on a cell type it infects.

Since even at high MOI RV infected HUVEC cultures continue to grow and multiply normally producing consistently high virus titers, co-adaptation of cells and virus was not necessary to establish persistent cultures, in which all cells were productively infected. Persistence of clinical wtRV strains in endothelial cells is sustained on a single cell level, i.e. the infected cells survive and transfer viral RNA or infectious virions into daughter cells after cell division.

The cell/virus cultures systems previously used to study RV persistence had dramatically different characteristics than that used here. Since infections of continuous cell lines (e.g. Vero cells) with lab strains at high MOI are highly cytopathic, low MOI infections and prolonged serial passage were used to reach an equilibrium between rates of cell proliferation and cell killing; less cytopathic mutants persisted thereafter [21,54,57,58]. In primary cultures, low MOI infections alone were usually sufficient to establish persistence of lab strains because of less CPE [20,22,43,44]. The establishment of persistence of a lab strain in both cell lines and primary cultures was typically accompanied by cycling of virus titers. When equilibrium was reached, only a portion of cells were infected, releasing 2-3 logs fewer viral particles than acutely infected cultures. It was suggested that rubella persistence in the previously studied cell types was primarily sustained by a carrier culture mechanism. Only a fraction of cells in a carrier culture harbors infectious virus and uninfected cells proliferation is in balance with death of infected cells and virus spread [54,57] allowing the virus to persist on a population level.

Interestingly, we did not observe large fluctuations of virus titers in HUVEC persistently infected with lab strain F-Therien. Because the input and persistent wt viruses were identical in the HUVEC system, some characteristic(s) of HUVEC likely explains the lack of oscillation in virus titers. One possible characteristic of the HUVEC cultures resulting in no oscillation would be a very low but stable sensitivity of the culture to F-Therein (2-5% of cells infected at any given time). Some other low cytocidal viruses, such as coxsackievirus B (CVB) or CMV, can persist in endothelial cell cultures by a carrier culture mechanism [59–62]. Similar to F-Therein, CVB type 5 can persist in endothelial cells by infecting 2% of cells in chronic phase and producing consistent virus titers [60]. Many other viruses can persistently infect humans and cause disease. However, the characteristics of persistence can be very different than that seen in CRS. For example, measles virus persists in SSPE cases, but in general the persistent state is maintained in the absence of infectious virus [63,64].

Fetal vascular defects can be caused by a number of factors including genetic disorders, environmental factors, or congenital infections. However, it was noted that vascular pathologies induced by congenital rubella in large blood vessels are unique and consist of lesions that occurred in the inner layer of vascular wall (intima), which otherwise are structurally preserved without necrosis, calcification or inflammation [8,14]. Our finding that RV does not cause cytopathic and ultrastructural changes in endothelial cells correlates well with this lack of structural damage in fetal vasculature. Microscopically, vascular lesions in CRS patients were described as extensive local proliferation of intima typically found near vascular branching points [8,13,14,65]. It is presently unknown what cell type proliferates and forms these vascular lesions: endothelial cells, which solely constitute intima, or cells transmigrated from other layers of vascular wall (e.g. smooth muscle cells). Although we did not observe enhanced proliferation of endothelial cells in response to infection in the static cell culture, RV infected cells might behave differently under conditions of hemodynamic shear stress. In the cell culture models of atherosclerosis, stimulation of endothelial cell proliferation was shown to occur in response to reduced hemodynamic shear stress and turbulent flow, the conditions found near branching points and vessel curvature [66,67]. Thus, an investigation of growth characteristics of infected HUVEC under various flow conditions might provide some insight into the role of RV infection in lesion formation. A possible role for smooth muscle cells in this pathological process should be explored as well.

CMV can also infect and persist in a fetus and cause an array of congenital defects; many of them are similar to those in CRS patients (e.g., deafness, growth retardation) [68,69]. However, unlike RV, CMV does not induce vascular pathologies in a fetus despite the fact that it has an ability to infect and persist in endothelial cells [70–72]. On the other hand, CMV was implicated in playing a role in acceleration of several vascular diseases in adults such as atherosclerosis, restenosis, and transplant vascular sclerosis. Chronic perivascular inflammation was shown to be responsible for the pathological process leading to vascular lesion formation in these diseases [73–75]. Since inflammation does not play a role in vascular lesion development in RV-infected fetuses, we think that molecular mechanisms of RV-induced vascular disease might be completely different from those occurring with CMV.

In conclusion, this study clearly shows that fetal endothelial cells are highly susceptible to rubella virus and support persistent virus infection providing more evidence for the suggestion that vascular pathologies in CRS involve persistent rubella virus infection of the endothelium [8,13,14]. We propose that HUVEC primary culture persistently infected with clinical RV strains is a reasonable model to study molecular mechanisms of rubella persistence.
